# Consensus for the integrated management of hepatitis C in Portugal

**DOI:** 10.1186/1471-2334-14-S6-S9

**Published:** 2014-09-19

**Authors:** Ricardo Baptista Leite

**Affiliations:** 1 Faculty of Health, Medicine and Life Sciences, Maastricht University, Maastricht, the Netherlands; 2 Instituto de Ciências da Saúde, Universidade Católica Portuguesa, Lisboa, Portugal

## 

The important work carried out by the European Centre for Disease Control and Prevention and the World Health Organization has demonstrated that 15 million people live with hepatitis C and that some 86,000 people die due to complications of this infection in the European Region alone [1]. The scientific community has presented us with a paradigm shift on the treatment of hepatitis C, having transformed this chronic (and highly fatal) disease into a curable condition. On the other hand, the high costs of the drugs that are capable of achieving such lifesaving results are a true challenge for health budgets worldwide.

Therefore, the parliamentary meeting organised by the Hepatitis C European Initiative in Brussels in February 2014 was of the utmost importance. It brought together stakeholders from different Member States of the European Union to share experiences and knowledge, while also reinforcing the need for us to work as a network on solutions to the challenges we now face.

At the meeting, I took the opportunity to present the methodology of a study on hepatitis C that I was coordinating at the Catholic University of Portugal. We focused on generating a consensus paper, which was presented on 13 May under the title: “Strategic Consensus for the Integrated Management of Hepatitis C in Portugal.”

This consensus [2] is the result of a Portuguese “think tank” of around 30 people chosen by a steering committee, with representation from the realms of science, clinical practice, health management, politics and patients. The meeting and discussions were held between October 2013 and March 2014.

From prevention to treatment, we presented recommendations based on different levels of concurrence among think tank participants. The following recommendations achieved the highest level of agreement:

1. International estimates [3] state that the prevalence of hepatitis C in the Portuguese population is between 1% and 1.5% and that around 900 to 1,200 people per year may die due to hepatitis C complications such as cirrhosis, hepatocellular carcinoma, HIV co-infection and non-specific liver failure.

2. Direct costs associated with hepatitis C treatment in Portugal, mainly related to late stages of the disease, run up to 70 million euros per year.

3. It is urgent to do more regarding hepatitis C prevention. The control of the disease in the long run depends on the reduction of new infections. There must be an active reinforcement of risk reduction policies, health education, and measures that limit transmission (e.g., broadening the criteria for hepatitis C screening).

4. It is necessary to have a management and referral network based on scientific criteria and subject to periodic audits.

5. There is an urgent need for updated national protocols for clinical guidance, and for these to be constantly maintained in line with scientific developments. At the same time, their actual use should be verified via auditing processes.

6. The high costs associated with new therapeutic formulations require new financing and negotiation models (i.e., risk sharing). The aim is to ensure access to innovation based on clinical criteria of priorities and social justice, within the context of the Portugal National Health Service’s financing restrictions.

7. Hepatitis C disease notification (and centralized registration) must be mandatory so that patients can have access to the most adequate treatments.

8. The lack of up-to-date knowledge among healthcare professionals concerning hepatitis C (and other liver diseases) must be urgently corrected.

9. An integrated management system for hepatitis C disease has been shown to be the most appropriate one and therefore it is necessary to have a national action plan and clear leadership at a central level.

In conclusion, as a society we need to find innovative clinical, administrative and financial strategies to overcome the budgetary barriers that limit patient access to life-saving treatments. To do so in a successful manner will be a major contribution towards the sustainability of our national health systems.

## Competing interests

The authors declare that they have no competing interests.

## Declarations

This article has been published as part of *BMC Infectious Diseases* Volume 14 Supplement 6, 2014: Viral Hepatitis in Europe. The full contents of the supplement are available online at http://www.biomedcentral.com/bmcinfectdis/supplements/14/S6. The publication charges for this supplement were funded by AbbVie as an unrestricted grant to Rigshospitalet, the University of Copenhagen. AbbVie further funded the printing of the supplement with additional financial support from the Drug Prevention and Information Programme (DPIP) of the European Union.

## References

[B1] World Health Organization “Prevention and Control of Viral Hepatitis Infection: Framework for Global Action” 2012 Available at: http://www.who.int/csr/disease/hepatitis/GHP_framework.pdf (Last accessed 16 July 2014)

[B2] Baptista Leite R Lopes H Tato Marinho R Peixe P “Strategic Consensus for the Integrated Management of Hepatitis C in Portugal” 2014 Universidade Católica Editora http://www.consensohepatitec.pt

[B3] Esteban JI Sauleda S Quer J The changing epidemiology of hepatitis C virus infection in Europe Journal of Hepatology 48 2008 148 162 18022726 10.1016/j.jhep.2007.07.033

